# Spontaneous Osteoclastogenesis, a risk factor for bone metastasis in advanced luminal A-type breast cancer patients

**DOI:** 10.3389/fonc.2023.1073793

**Published:** 2023-02-20

**Authors:** Valeria Fernández Vallone, Francisco Raúl Borzone, Leandro Marcelo Martinez, María Belén Giorello, Hosoon Choi, Federico Dimase, Leonardo Feldman, Raúl Horacio Bordenave, Ana Marisa Chudzinski-Tavassi, Emilio Batagelj, Norma Alejandra Chasseing

**Affiliations:** ^1^ Berlin Institute of Health at Charité – Universitätsmedizin Berlin, Core Unit Pluripotent Stem Cells and Organoids, Berlin, Germany; ^2^ Laboratorio de Inmunohematología, Instituto de Biología y Medicina Experimental (IBYME), Consejo Nacional de Investigaciones Científicas y Técnicas (CONICET), Buenos Aires, Argentina; ^3^ Department of Medicine, Hematology and Medical Oncology, Weill Cornell Medical College, New York, NY, United States; ^4^ Research Service, Central Texas Veterans Health Care System, Temple, Texas, TX, United States; ^5^ Servicio de Hematología, Hospital Militar Central, Buenos Aires, Argentina; ^6^ Facultad de Ciencias de la Salud, Universidad Nacional del Centro de la Provincia de Buenos Aires (UNCPB), Tandil, Buenos Aires, Argentina; ^7^ Sala de Oncología, Hospital Iriarte, Quilmes, Buenos Aires, Argentina; ^8^ Laboratory of Development and Innovation/Center of Excellence in New Target Discovery, Instituto Butantan, São Paulo, Brazil; ^9^ Servicio de Oncología, Hospital Militar Central, Buenos Aires, Argentina

**Keywords:** pre-metastatic niche, bone metastasis, RANKL, CCL-2, spontaneous osteoclastogenesis

## Abstract

**Introduction:**

Osteolytic bone metastasis in advanced breast cancer stages are a major complication for patient´s quality life and a sign of low survival prognosis. Permissive microenvironments which allow cancer cell secondary homing and later proliferation are fundamental for metastatic processes. The causes and mechanisms behind bone metastasis in breast cancer patients are still an unsolved puzzle. Therefore, in this work we contribute to describe bone marrow pre-metastatic niche in advanced breast cancer patients.

**Results:**

We show an increase in osteoclasts precursors with a concomitant imbalance towards spontaneous osteoclastogenesis which can be evidenced at bone marrow and peripheral levels. Pro-osteoclastogenic factors RANKL and CCL-2 may contribute to bone resorption signature observed in bone marrow. Meanwhile, expression levels of specific microRNAs in primary breast tumors may already indicate a pro-osteoclastogenic scenario prior to bone metastasis.

**Discussion:**

The discovery of prognostic biomarkers and novel therapeutic targets linked to bone metastasis initiation and development are a promising perspective for preventive treatments and metastasis management in advanced breast cancer patients.

## Graphical Abstract

Graphical abstract describing the findings and the complexity of systemic inter-relationships between bone marrow and the primary breast tumor. Spontaneous osteoclastogenesis (spontaneous OC); mesenchymal stem/stromal cell (MSC).
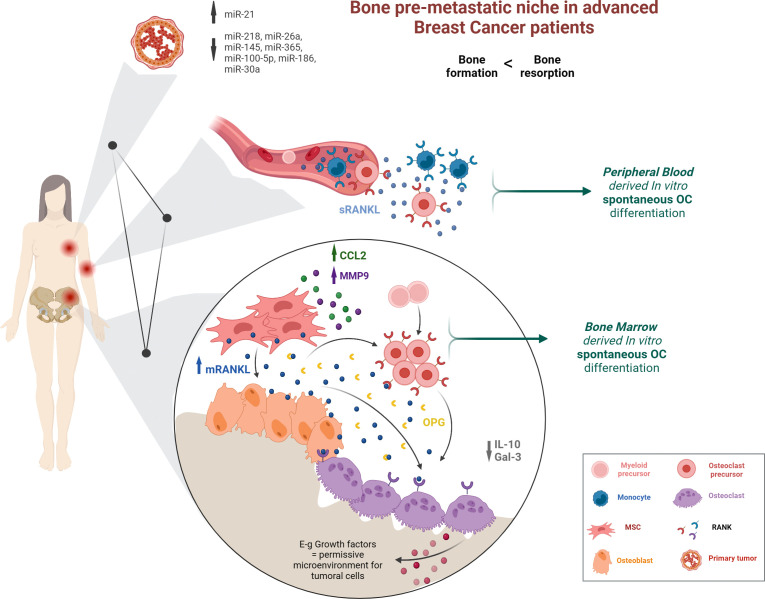

## Highlights

- Advanced luminal A-type BCPs prior to bone metastasis show SpOC when both BM and PB precursors are cultured *in vitro*.
- Advanced BCPs have higher number of RANK+ circulating OCPs.
- Key factors in BM such as RANKL and CCL-2 contribute to a pro-resorptive PMN.
- Primary tumor miR profile in advanced BCPs may indicate an osteoclastogenic bias.
- Understanding the complex interrelationship between primary tumor-circulation-BM would help to define better bone metastasis prognosis and BCP management.

## 1 Introduction

Over 65% of breast cancer patients (BCPs) with late clinical-pathological stage suffer from bone metastasis (1). Even though, bone metastasis can be a mix of osteoblastic (bone deposition) or osteolytic (bone destruction), it is the last type which appears more frequently in BCPs (2, 3). Osteolytic bone metastasis are very aggressive and severely affect patients outcome with complications such as fracture, bone pain, hypercalcemia and spinal cord compression (2). Bone metastasis initiation process is not well understood, circulating cancer cells should enter bone marrow (BM) microenvironment and occupy the hematopoietic niche, where most of the times remain dormant (and comfortable) for a long period of time until they reactivate by increasing proliferation leading to micrometastasis (2). A century ago, Stephen Paget made the first known mention to the concept of pre-metastatic niche (PMN) in his “Seed and Soil” theory (4). Tumor cells (seeds) can colonize and grow in permissive microenviroments (soil) with nutrients, a remodeled extracellular matrix and supportive signals from the different stromal cells; backing altogether the metastatic process (5). Furthermore, in two-thirds of the patients, metastasis will not be limited to the skeleton, but rather later occur to other organs and eventually cause death (6, 7). Therefore, understanding the BM/bone PMN and the chain of events which enable circulating cancer cells to home in perivascular and endostio niches are of capital importance in order to prevent or stop the metastatic multi-step process. The discovery of new therapeutic targets which could avoid end-stage multi-organ metastasis, that cause the vast majority of BCP deaths, is an unmet clinical need.

Bone physiology in healthy individuals undergoes continual remodeling to grow, heal damage, and regulate calcium and phosphate metabolism (6). This process requires a tight and continuous balance between bone formation, in charge of osteoblasts, and bone resorption, orchestrated by osteoclasts (OCs) (3, 7). BM mesenchymal stem/stromal cells (MSCs) are osteoblast progenitors, secrete bone matrix proteins for bone formation and final osteocyte differentiation (2). MSCs and osteoblasts produce the canonical osteoclastogenic factors, receptor activator of nuclear factor kappa B ligand (RANKL) and macrophage colony-stimulating factor (M-CSF) which promote mielo-monocytic progenitors, monocytes and macrophages differentiation into OC based on their phagocytic and fusion capacity (8–12). In parallel, MSCs and osteoblasts secrete the main OC differentiation inhibitor decoy receptor osteoprotegerin (OPG) (10). Therefore, MSCs and osteoblasts regulate bone resorption process. At the same time, mature OC either by clastokine secretion, cell to cell contact or through enzymatic activity degrading bone matrix and releasing cytokines [e.g., insulin-like growth factor-1 (IGF-1), transforming growth factor beta (TGF-ß)], stimulate osteoblast survival and differentiation (6). Accordingly, osteoblast-OC interactions are considered a basic multicellular unit (6). Correct function of this unit in BM is crucial for controlling blood and bone formation. Aberrant expression of signaling molecules, improper differentiation processes or cytokines secretion can facilitate tumor cell colonization and outgrowth in the bone. Once in the bone, cancer cells secrete pro-OC maturation factors [e.g. parathormone (PTH), interleukin (IL)-11, tumor necrosis factor alpha (TNF-α)] which stimulate osteoblasts to increase RANKL and decrease OPG production, feeding a `vicious cycle´ (13).

We have previously shown that a selected BCPs group defined as untreated, late stage without detectable BM/bone metastasis or osteoporosis disease, had lower number of MSCs in BM compared to healthy volunteers (HVs) (14). In addition, BM-MSCs from BCPs had low osteogenic and adipogenic differentiation capacity (15). Both observations, suggested an altered BM niche, with an imbalanced bone metabolism, displaying the importance of microenvironment regulation by MSCs. We, therefore, propose the BM of advanced BCPs, to be a permissive niche for cancer cell homing. In the present work and primed by the coupling theory which implies that OC `talk back´ to cells of the osteoblast linage, we decided to evaluate the other side of the coin regarding bone imbalance: OC differentiation capacity and maturation in BM and peripheral blood (PB) from BCPs.

Our findings indicate an active spontaneous OC differentiation (SpOC) process in BCPs which can be evident in BM and PB. We have identified a pre-activated state of BM-mononuclear cells (MNCs) and PB monocytes compatible with OC precursor (OCP) state based on surface markers. We show that BCPs-BM has shifted to a pro-osteoclastogenic microenvironment as MSCs highly express RANKL, matrix metalloproteinase 9 (MMP-9) and C-C motif chemokine ligand 2 (CCL-2) before the evidence of any bone/BM metastasis. Accordingly, the primary tumor remotely contributes to prepare BM niche for future metastasis. We give an example of such contribution by studying microRNAs (miRs) expression levels in primary breast tumor which have been recently shown to mediate different aspects of bone biology by gene regulation (16–18). We finally propose promising biomarkers which may help to reveal an early bone metabolism unbalance which should be follow up for early bone metastasis prognosis and management.

## 2 Materials and methods

### 2.1 Patients

We conducted a prospective study including 14 patients diagnosed with breast cancer confirmed by histology and 10 HVs as controls. Inclusion criteria was women with advanced invasive ductal breast carcinoma (stage III), according to the TNM classification system of the International Union Against Cancer. Exclusion criteria were neoadjuvant therapies and previous breast tumor surgery, another primary tumor development, presence of BM and/or bone metastasis and osteoporosis, as well as metabolic bone disease, such as vitamin D deficiency, thyroid disease, parathyroid disease or kidney damage. All BCP tumors were classified as Luminal A [estrogen receptor (ER) +, progesterone receptor (PR) +, epidermal growth factor receptor (HER2/neu (-)]. In addition, the patients were in menopause with an age range from 50 to 65 years old. Inclusion criteria for the HV group was to be a female donor for bone marrow transplant in menopause and exclusion criteria were female donors with osteoporosis, or metabolic bone disease, such as vitamin D deficiency, thyroid disease, parathyroid disease or kidney damage. HV had an age range between 45–65 years old. All individuals gave consent to participate in this study, which was performed in accordance with the principles of the Helsinki Declaration. IBYME-Ethical Committee approved this investigation and informed consent.

### 2.2 Bone and BM metastasis assessment

The absence of bone metastasis was confirmed by X-ray and bone scintigraphy. Neoplastic cells infiltration in BM aspirates was ruled out by immunocytochemistry staining (Universal LSAB System, K0673, Dako) using monoclonal antibodies (Abs) against epithelial membrane antigen (IS629, Dako) and cytokeratin AE1–AE3 (IR053, Dako). Cell morphology analysis was performed by the Pappenheim technique. Patient´s BM were considered positive for metastasis if cells expressed epithelial membrane antigen and cytokeratins AE1–AE3 and were morphologically malignant.

### 2.3 BM plasma collection and BM-MNCs isolation

For BM collection, BCPs and HVs were positioned in lateral decubitus position, and local anesthetic solution (1% plain lidocaine) was placed around the harvest site. Then a harvest needle with a sharp trocar was introduced percutaneously through the skin until posterior iliac crest. Subsequently, the sharp trocar was pulled out and at that point 10 ml of BM were aspirated using a 20 ml plastic syringe attached to the aspiration needle and collected in heparinized saline without preservatives (25 units/ml, 15077-019, Gibco). 3 ml from each aspirate was centrifuged at 1,125 g for 45 minutes (min) at 4°C. The plasma obtained was aliquoted and stored at -20°C until use. The rest of the sample (~7 ml) was diluted 1 to 2 with phosphate-buffered saline (PBS) and slowly layered on Histopaque (density = 1,075 gr/cm^3^, H8889, Sigma) (19). After 25 min centrifugation at 340 g, the gradient interface containing BM light density MNCs was carefully collected, washed twice in PBS and re-suspended in supplemented α-minimal essential medium (α-MEM, cat. 11900024, Gibco) containing 2 mM L-glutamine (25030081, Gibco), 100 IU/ml Antibiotic-Antimycotic (15240062, Gibco) [*supplemented α-MEM*] and 20% heat-inactivated fetal bovine serum (FBS, 16000044, Gibco). MNCs suspension was counted using 3% acetic acid solution (2000165308, Biopack). Cell viability was determined by 0.04% trypan blue dye exclusion (T 0887, Sigma).

### 2.4 Osteoclastogenic differentiation of MNCs from BM

Viable MNCs from 6 BCPs and 8 HVs were isolated, as above described, and seeded in 8 well chamber Slide (177445, LabTek, Nunc) at 1 x 10^6^ MNCs per well. Osteoclastogenic differentiation of MNCs from BM was performed taking into account the methods of Koshihara Y et al. and Baek KH et al. with modifications (19, 20). Briefly, cells were cultured in supplemented α-MEM and 20% horse serum (16050130, Gibco) for 5 days. After this period, non-adherent cells were removed, adherent cells were washed one time with PBS and medium was renewed. Adherent cells were cultured in supplemented α-MEM and 20% horse serum, with or without 10^-8^ M 1α, 25-Dihydroxyvitamin D3 (VitD3, D1530, Sigma) for a total of 18 days, renewing 50% of the medium every 3 days. OCs were identified using the Acid Phosphatase, Leukocyte [TRAP (tartrate-resistant acid phosphatase) Kit, 387A, Sigma] according to manufacturer instructions. Cells nuclei were counterstained with hematoxylin. Cells were visualized and photographed with an inverted phase-contrast Olympus CKX41 microscope using Olympus camera Q-Color 5. TRAP-positive cells with more than 3 nuclei were counted as OC. The number of OCs per 200 cells by well were counted and expressed as media. Nuclei per OC, and planar area were cytomorphometrically determined as number of nuclei and area per OC measured using ImageJ analysis software. Experiments were repeated two times for each individual sample.

### 2.5 PB plasma collection

PB samples from 5 BCPs and 6 HVs were collected into heparinized saline without preservatives (25 units/ml, 15077-019, Gibco). An aliquot of 5 ml was centrifuged at 1,125 g for 45 min at 4°C. The PB plasma obtained was aliquoted and stored at -20°C until use.

### 2.6 Osteoclastogenic differentiation of monocytes (CD14 +) from PB

Monocytes were isolated from the remaining whole blood (~10 ml) using RosetteSep™ Human Monocyte Enrichment Cocktail (negative selection kit, 15028, STEMCELL Technologies Inc.) following manufacturer´s instructions. After 20 min of incubation with the cocktail (tetrameric Antibody Complexes (TAC) that recognizes non-monocyte cells and red blood cells), samples were half diluted with PBS-2% FBS and layered on Histopaque. The gradient was centrifuged for 20 min at 1,125 g. The interface, enriched in monocytes, was harvested, washed twice in PBS and re-suspended in supplemented α-MEM. Cell suspension was counted using 3% acetic acid solution and cell viability was determined by 0.04% trypan blue dye exclusion. PB monocytes (CD14 +) differentiation into OC were performed taking into account the methods described by Roato et al, Komano et al, and Sørensen et al. with modifications (21–24). Briefly, viable MNCs enriched in monocytes CD14+, from 5 BCPs and 6 HVs were seeded in 8 well chamber, 2 x 10^5^ cells per well, containing 500 µl of supplemented α-MEM with 10% FBS and 25 ng/ml of human recombinant M-CSF factor (hrM-CSF; GF053, Millipore). After 3 days, non-adherent cells were removed, and the adherent cells were washed one time with PBS and cultured in supplemented α-MEM, with 10% FBS and 25 ng/ml hrM-CSF, in the presence or not of 25 ng/ml human recombinant RANKL (hrRANKL; GF091, Millipore) for a total of 18 days. During the differentiation, the medium was renewed every 3 days. At the end, cells were stained using TRAP Kit and cell nuclei counterstained with hematoxylin. Cells were visualized and photographed with an inverted phase-contrast Olympus CKX41 microscope using Olympus camera Q-Color 5. TRAP positive cells with more than 3 nuclei were counted as OC. The number of OCs per 200 cells by well were counted and expressed as media. Nuclei per OC and planar area were cytomorphometrically determined as number of nuclei and area per OC measured using ImageJ analysis software. Experiments were repeated two times for each individual sample.

### 2.7 BM and PB derived OCs characterization

In order to study specific mature OC markers, the above described differentiation protocols were performed on bovine cortical bone slices (BON1001, Nordic Biosciences). Briefly, 1 x 10^6^ isolated BM-MNCs (6 BCPs and 8 HVs) and 2 x 10^5^ PB monocytes-CD14+ (5 BCPs and 6 HVs) were seeded on a bone slice placed in one well of 16 well chamber Slide (178599, LabTek, Nunc) and cultured for 18 days.

#### 2.7.1 Immunohistochemistry

At the end of the culture period, OCs were fixated in 4% paraformaldehyde (158127, Sigma) in PBS for 20 min at room temperature (RT) and then wash in PBS. Thereafter, endogenous peroxidase was blocked by incubating the sample for 5 min in 3% hydrogen peroxide, followed by 2 washes with Tris-buffered saline (TBS, (Tris, 161-0716, BioRad and NaCl, 1.06404, Merck)). Samples were permeabilized using 0.1% Triton X-100 (X100, Sigma) in TBS for 30 min at RT. After two washes in 0.1% Tween-20 (T20, 20605, USB Corp), protein block was performed with 1% bovine serum albumin (BSA) in 0.1% T20-TBS (BSA-T20-TBS) for one hour at RT. This step was followed by overnight 4°C primary Abs incubation: MMP-9 (mouse IgG1, MAB911, R&D Systems), c-Src kinase (mouse IgG2a, MAB3807, R&D Systems) and CD51 (goat IgG, AF1219, R&D Systems), all diluted in the blocking solution. Corresponding isotype control Abs were used at same concentration of each primary Ab tested. Isotype controls: mouse IgG (MAB002, R&D system), mouse IgG2a (MAB003, R&D system) and goat IgG (AB-108-C, R&D Systems). After overnight incubation, samples were washed 3 times with TBS followed by immunocytochemistry detection using LSAB+ System-HRP (K0690, Dako) and 3-3’-diaminobenzidine (Liquid DAB, Substrate Chromogen System, K3468, Dako) according to manufacturer instructions. Cells were visualized, by turning the bovine cortical bone slices up side-down, and photographed using an inverted phase-contrast Olympus CKX41 microscope equipped with Olympus camera Q-Color 5. Each marker studied was carried out by duplicate.

Bone resorption lacunae formation and OC acidification capacity were used as functional study parameters. Both assays were performed on BM-derived (6 BCPs and 8 HVs) and PB-derived (5 BCPs and 6 HVs) OC. Each study was carried out by duplicate.

#### 2.7.2 Bone resorption lacunae formation

OC differentiation was performed on bovine cortical slices as described before. After 18 days of culture, bone slices were washed in water, and incubated overnight with 2 N sodium hydroxide (6498, Merck) at 4°C. Resorption pits were identified using 1% toluidine blue staining (89640, Fluka) in 0.5 M sodium tetraborate solution (59640, Sigma) for 3 min at RT. Excess staining was removed by washing in distilled water and then air-drying. Toluidine Blue stains the edge of the bone resorption zone left by the OC which is no longer present after sodium hydroxide lysis. Resorption lacunae visualization was carried out under an inverted phase-contrast Olympus CKX41 microscope with 10×/0.25 objective by turning the bovine cortical bone slices upside-down.

#### 2.7.3 Osteoclast acidification capacity assay

Differentiation protocol was performed in a 16 well chamber glass (17599, LabTek, NUNC). At day 18, acridine orange (318337, Sigma) was added to the culture medium at a final concentration of 10 µg/ml, for 45min, in the dark, at RT, after this, cells were carefully washed and maintained in PBS for immediate visualization. The cells were viewed and photographed with an inverted phase-contrast Olympus CKX41 microscope equipped with 510/540 nm excitation filter using Olympus camera Q-Color 5.

### 2.8 Phenotypic characterization of OCPs from BM and PB by FACS

BM-MNCs from 5 BCPs and 8 HVs, as well as PB-MNCs from 5 BCPs and 5 HVs, were collected and isolated as described above. Viable MNCs (5 x 10^5^) were incubated in 5% AB serum (S7148, Sigma) diluted in PBS for 30 min at 4°C, to reduce non-specific binding. Single labelling using primary Abs anti-human: anti-RANK (RANKL receptor, mouse IgG1, MAB683, R&D Systems), anti-CD11b-PE (mouse IgG2ak, 347557, BD Bioscience), anti-CD14 (mouse IgG1k, 14-0149, eBioscience), anti- CD51/61-FITC (integrin alphaV/beta3, mouse IgG1k, 555505, BD Bioscience) and anti-CD115 (or c-fms, M-CSF receptor, rat IgG1, 4-1159, eBioscience) were performed. For uncoupled Abs an incubation with secondary Ab FITC-conjugated (anti mouse-IgG, 115-095-164, Jackson Inmuno Research) or PE-conjugated (anti rat-IgG-PE, F0105B, R&D Systems) followed. Isotype controls were run in parallel using the same concentration of each primary Ab tested. Isotype controls: mouse IgG1 (MAB002, R&D system), mouse IgG2ak-PE (556653, BD Bioscience), mouse IgG1k (ab91353, abcam), mouse IgG1k-FITC (551954, BD Bioscience) and rat IgG1 (14-4301-82, eBiosciences). After labelling, cells were washed using 3% BSA (A7906, Sigma) in PBS (BSA-PBS). Thereafter, cells were fixed in 1% formaldehyde (1044, Cicarelli Corp.) in PBS for 20 min at 4°C. At least 10,000 events were acquired using FACScalibur (BD Biosciences). FlowJo X v10.0.7 (FlowJo, LLC) software was used to create the histograms and density plots. Results were expressed as percentage and relative fluorescence index (RFI= marker mean fluorescence index/corresponding isotype control mean fluorescence index). Experiments were repeated two times for each individual sample.

### 2.9 Primary culture of MSCs

MNCs (10 x 10^6^) obtained from BM of 6 BCPs and 5 HVs were plated in 25 cm^2^ tissue culture flasks with 10 ml of supplemented α-MEM containing 20% FBS. Cells were incubated at 37°C, 5% CO_2_ and humidified environment. After 24 hs, non-adherent cells were removed, and the medium was renewed. Cells were cultured until they reached 70-80% confluence, renewing the medium every 7 days. Conditioned media (CM) from last 7 days were collected, centrifuged at 250 g for 10 min and the supernatant transferred to a fresh tube and frozen at -20°C until use. Then MSCs were washed twice with PBS and harvested with a solution of 0.05% trypsin-EDTA (15400054, Gibco) in PBS for further use.

#### 2.9.1 RANKL secretion

RANKL levels in CM were measured using a commercial ELISA Kit (RHF740CKC, Antigenix America) according to the manufacturer’s recommendations. Each sample was assessed in triplicate.

#### 2.9.2 Membrane RANKL (mRANKL) expression

Membrane RANKL (mRANKL) expression was assessed on viable MSCs by FACS using a primary anti-human RANKL-Ab (rabbit IgG, AB1862, CHEMICON) and a secondary FITC conjugated Ab (donkey IgG, NL006, R&D Systems). A related isotype control was used (rabbit IgG, AB-105-C, RD System). In addition to this, mRANKL was also assessed by immunocytochemistry. Briefly, viable BM-derived MNCs (4 BCPs and 5 HVs) were seeded at 3 x10^4^ cells per well on Permanox™ solvent-resistant plastic chamber slides (177445, LabTek, Nunc) and cultured in supplemented α-MEM containing 20% of FBS. After 24 hs, non-adherent cells were removed and the medium was renewed. Primary cultures were incubated until they reached 70-80% of confluence, renewing the medium every 7 days. MSCs were washed twice with PBS and once in 0.1% T20-TBS. Thereafter, endogenous peroxidase was blocked by 5 min incubation in 3% hydrogen peroxide, followed by 3 washes with 0.1% T20-TBS. Non-specific protein binding sites were blocked by 1 hour incubation in 1% BSA-0.1% T20-TBS at RT. Primary Abs against human RANKL (rabbit IgG, AB1862, CHEMICON), prolyl-4-hydroxylase (mouse IgG1 K, M0877, DAKO) and anti-CD68 (mouse IgG1 K, M0718, DAKO), were diluted in BSA-T20-TBS and incubated overnight at 4°C. Related isotype control, rabbit IgG (AB-105-C, RD System), mouse IgG1 (X0931, Dako) as well as a negative control (normal mouse total IgG and IgM, 08-6599, ZYMED) were performed in parallel. Universal biotin-Streptavidin-HRP detection system (K0679, Dako) was used. Antigen detection was visualized using diamino benzidine (DAB, Dako). Percentage of positive cells (number of cells with brown granulations/200 total cells) was scored using a light microscope. Expression levels per cell were established qualitatively: + to ++++ with respect to two positive controls: one high (++++, β subunit of prolyl 4-hydroxylase) and one low (+, CD68). β subunit of prolyl 4-hydroxylase is present in 100% of the BM-MSCs (fibroblast-like) and CD68 is positive in approximately 60-70% of these cells. Experiments of both studies were repeated two times for each individual sample.

#### 2.9.3 Total RANKL expression

This factor in MSCs (6 BCPs and 5 HVs) was analyzed by Western Blot. Briefly, cells were lysed in RIPA buffer [Tris (pH=7.5) 25 mM, NaCl 360 mM, nonidet P-40 2.5%, sodium deoxycholate 1%, SDS 0.25%, sodium vanadate 5 mM], supplemented with proteases and phosphatases inhibitor cocktail (P8340, Sigma) at 4°C for 30 min. After centrifugation for 30 min at 16,000 g at 4°C, pellets were frozen and conserved at -80°C until use. SDS-PAGE electrophoresis was performed using 20 µg of denatured protein, later transferred onto a nitrocellulose membrane (RPN3032D, Amersham). The membrane was stained with 0.2% Ponceau S in 0.5% acetic acid to verify the integrity of the transferred proteins. After blocking with 5% skim milk in T20-TBS, the membranes were incubated with primary Abs against human RANKL (1:200, mouse IgG2b, MAB626, Clone: 70525, R&D Systems) and β-Actin (1:2000, mouse IgG1, ab6276, Clone: AC-15, Abcam). Goat anti mouse Ig conjugated to horseradish peroxidase was used as secondary Ab (goat IgG, HAF007, R&D Systems). Hybridizing bands were visualized using ECL™ Prime Western Blotting System (GERPN2232, Sigma) and were digitalized using the image analysis system (G Box Chemi XT16, Syngene, USA). Relative RANKL quantification was performed by normalization with β-Actin signal and expressed as mean relative density using ImageJ analysis software (NIH).

### 2.10 Evaluation of pro-osteoclastogenic and anti-osteoclastogenic factors in PB and BM plasma and CM of fibroblast colony-forming unit (CFU-F)

CFU-F assay was performed as we have previously described (15). Briefly, BM-derived MNCs from 14 BCPs and 10 HVs were cultured for 14 days and CM from last 7 days was collected centrifuged at 250 g for 10 min and supernatant transferred to a fresh tube and frozen at −20°C until use. Levels of the pro-osteoclastogenic factors IL-7, CCL-2 and M-CSF, as well as the anti-osteoclastogenic factors CCL-5 and Galectin-3 (Gal-3) were measured in PB and BM plasma and CM of CFU-F using ELISA kits: DY207 (R&D Systems), DY279 (R&D Systems), CK100233 (Antigenix America), DY278 (R&D Systems) and Dgal-30 (R&D Systems), respectively.

Moreover, levels of others pro–and anti-osteoclastogenic factors were quantified in PB and BM plasma using ELISA Kits: IL-1β (DLB50, R&D Systems), IL-17 (D1700, R&D Systems), TNF-α (DY210, R&D Systems) and granulocyte-macrophage colony-stimulating factor (G-CSF, DGM00, R&D Systems), IL-4 (DY050, R&D Systems), IL-10 (D1000, R&D Systems), interferon gamma (INF-γ, DIF50, R&D Systems), respectively. MMP-9 levels, pro- and activated-form, were evaluated in CM of CFU-F using Amersham Biosciences (RPN2614) and Amersham Biosciences (RPN2634) ELISA Kits. In addition, levels of others pro-osteoclastogenic factors were measured in CM of CFU-F using ELISA kits: IL-6 (DY206, R&D Systems), IL-8 (DY208, R&D Systems) and IL-11 (DY218, R&D Systems). In all the cases the assay was performed according to the manufacturer’s recommendations. Each sample quantification was performed in triplicate. In addition, CCL-2 levels were quantified in CM collected from MSCs primary cultures, as described above.

### 2.11 Functional evaluation of pro-osteoclastogenic MMP-9

The proteolytic activity of MMP-9 was evaluated in CM from CFU-F assay (14 BCPs and 10 HVs) by gelatin zymography assay. The concentration of total protein in CFU-F CM were quantified using Bradford reagent (25). Same protein concentration was adjusted (1 µg/µl) in all samples and mixed with 5X non-reducing sample buffer [20% glycerol, 0.01% bromophenol blue, 125 mM Tris-HCl, pH 6.8] and separated by electrophoresis (110V) using 7.5% polyacrylamide gel containing 0.1% gelatin. After electrophoresis gels were washed twice for 30 min in washing buffer (50 mM TrisHCl [pH 7.5], 2.5% Triton X-100, 5 mM CaCl2, 1 µM ZnCl2) at RT and incubated overnight at 37°C in incubation buffer (1% Triton X-100, 50 mM TrisHCl [pH 7.5], 5mM CaCl2, 1 µM ZnCl2). Gels were then stained for 30 min in staining buffer (40% methanol, 10% acetic acid, 0.5% Coomassie Brilliant Blue R-250) and then incubated with de-staining solution (40% methanol, 10% acetic acid) until bands could clearly be seen. Enzymatically active MMPs were visualized as clear bands against a blue background in the gel, indicating proteolysis of the substrate protein gelatin, and digitalized using the image analysis system (G Box Chemi XT16, Syngene, USA). The molecular mass of pro-MMP-9 and active-MMP-9 detected by zymograms were determined by comparison with reference protein molecular mass markers. As positive control CM collected from PC-3 cell line cultures (ATCC, Cat. CRL-1435) was used, a well-known producer of MMP-9. Unspecific gelatinolytic action was discarded using as negative control α-supplemented media containing 20% FBS incubated for 7 days under same conditions as the cultures of interest. The density of the lysis band was normalized to the density value of the positive control and expressed as arbitrary units/ml, using ImageJ.

### 2.12 Analysis of TCGA dataset

Mature miRs expression in primary tumors of BCPs was obtained from the TCGA Breast Cancer (BRCA) cohort available in the UCSC Xena bioinformatics tool (26). Mature miR strand expression RNAseq (IlluminaHiSeq) data from 16 BCPs (invasive ductal breast carcinoma, clinical stage III-A, -B and –C, luminal type A) samples paired with 56 normal breast tissues were included in the analysis. We evaluated a pool of miRs involved in the *regulation of monocyte differentiation to pre-OC*: miR-21 (MIMAT0000076), miR-29 family (MIMAT0000086), miR-100-5p (MIMAT0000098), miR -26a (MIMAT0000082), miR-125a (MIMAT0000443), miR-145 (MIMAT0000437), miR-214 (MIMAT0000271), miR-218 (MIMAT0000275), miR-301b (MIMAT0004958) and miR-365 (MIMAT0000710); *fusion of pre-OC to inactive OC*: miR-7b (MIMAT0004553) and miR-30a (MIMAT0000087) and *regulation of OC function, survival and apoptosis:* miR-17 (MIMAT0000070), miR-106b (MIMAT0000680), miR-27a (MIMAT0000084), miR-29b (MIMAT0000100), miR-31 (MIMAT0000089), miR-146a (MIMAT0000449), miR-155 (MIMAT0000646), miR-186 (MIMAT0000456), miR-193-3p (MIMAT0000459) and miR-539 (MIMAT0003163).

### 2.13 Statistical test

Results were expressed as mean ± standard error (SEM). Statistical analysis was performed using GraphPad Prism 5.0 software (GraphPad Software Inc., CA, USA). Correspondent parametric and non-parametric tests were carried out as described in results. Differences were considered statistically significant when p < 0.05.

## 3 Results

### 3.1 BM-MNCs and PB monocytes from BCPs spontaneously differentiate into functional and mature OCs

**Figure 1:**
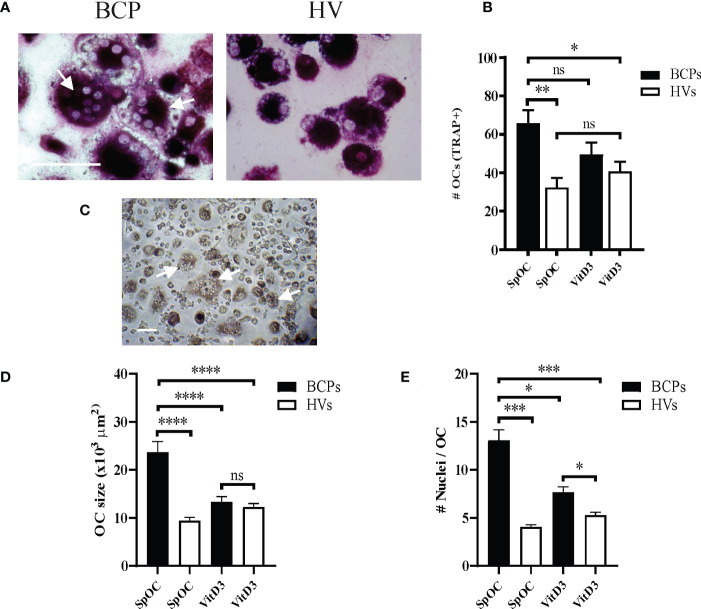
Bone marrow (BM) mononuclear cells (MNCs) from breast cancer patients (BCPs) spontaneously differentiate into osteoclasts (OCs). **(A)** Representative pictures showing TRAP staining performed on spontaneously differentiated OC (SpOC) derived from a BCP or a healthy volunteer (HV) BM- MNCs at day 18. Arrows show TRAP+ multinucleated OC. Scale bar: 100 µm. **(B)** Quantification number of OCs (TRAP+) is shown as mean ± SEM. BCPs, n=6 and HVs, n=8. Statistical analysis: One-way ANOVA followed by Tukey post hoc test; p<0.050 (*); p<0.010 (**); ns, not significative. **(C)** Example of BCP SpOC culture at day 5. Arrows show multinucleated OC. Scale bar: 100 µm. **(D)** Average OC planar area (size in μm2). Data are shown as mean ± SEM. BCPs, n=4 and HVs, n=4. Statistical analysis: One-way ANOVA followed by Tukey post hoc test; p<0.0001 (****). **(E)** Nuclei number per OC. Data are shown as mean ± SEM. BCPs, n=4 and HVs, n=4. Statistical analysis: Kruskal–Wallis test followed by a Dunn’s Multiple Comparison test; p<0.029 (*) and p<0.0001 (***).

BM-MNCs from BCPs and HVs were differentiated into OCs in the absence of VitD3 to explore their spontaneous osteoclastogenic capacity. After 18 days of culture, SpOC was observed in both groups (**Figure 1A**; **Supplementary Figure 1A**). OCs showed expected morphology and characteristics of mature OC such as dome shape, basophilic cytoplasm, multiple rounded nuclei and positive acid phosphatase activity evidenced as purplish to dark red granules in the cytoplasm (**Figure 1A**). SpOC differentiation assessed by number of TRAP+ OCs was significantly higher in BCPs compared to HVs cultures (X ± SEM= 65.85 ± 6.81 and 32.35 ± 5.00, respectively) (**Figure 1B**). Of note, early as day 5 of culture and in the absence of VitD3, BM-MNCs isolated from BCPs, showed appearance of multinucleated cells compatible with OCs which were yet not seen in HVs cultures (**Figure 1C**).

VitD3 supplementation during culture slightly enhanced OC differentiation efficiency in HVs samples when compared to SpOC (OC number increase percentage 25.87). On the contrary, VitD3 appear not to affect or even decrease OC differentiation efficiency in BCPs cultures with respect to SpOC (**Figure 1B**). Interestingly, BCPs cultures with SpOC not only showed the highest differentiation efficiency but also the larger OC with the greater nuclei number, even when compared to VitD3 supplemented HVs cultures (**Figures 1D, E**). In line with these observations, BCPs cultures with SpOC presented a greater number of OCP, visualized as cells with a central nucleus and positive TRAP staining, and fusion processes depicting faster OC maturation (**Figure 1A**; **Supplementary Figure 1A**).

**Figure 2:**
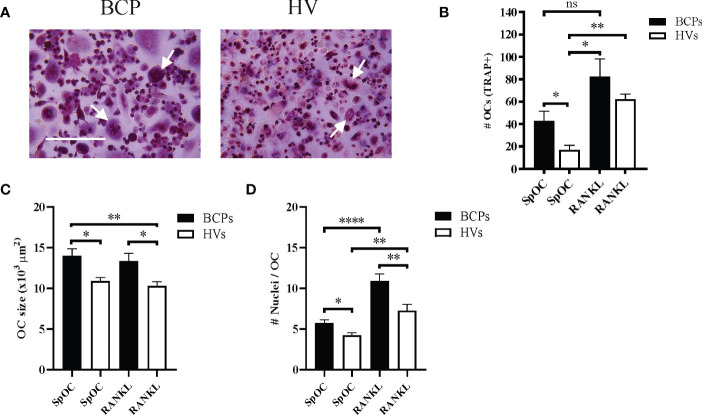
Peripheral blood CD14+ monocytes from breast cancer patients (BCPs) spontaneously differentiate into osteoclasts (OCs). **(A)** Representative pictures showing TRAP staining performed on spontaneously differentiated OC (SpOC) CD14+ monocytes from a BCP or healthy volunteer (HV) at day 18. Arrows show multinucleated TRAP+ OC. Scale bars: 100 µm. **(B)** Quantification number of OCs (TRAP+) is shown as mean ± SEM. BCPs, n=4 and HVs, n=6. Statistical analysis: Kruskal–Wallis test followed by a Dunn’s Multiple Comparison test; p <0.050 (*); p<0.010(**); ns, not significative. **(C)** Average OC planar area (size in μm2). Data are shown as mean ± SEM. BCPs, n=4 and HVs, n=4. Statistical analysis: Kruskal–Wallis test followed by a Dunn’s Multiple Comparison test; p <0.050 (*) and p<0.010(**). **(D)** Nuclei number per OC. Data are shown as mean ± SEM. BCPs, n=4 and HVs, n=4. Statistical analysis: Kruskal–Wallis test followed by a Dunn’s Multiple Comparison test; p <0.050 (*), p<0.010(**) and p<0.0001(****).

Classical *in vitro* OC differentiation protocols using CD14+ monocytes isolated from PB rely on M-CSF and RANKL presence in the culture media (24). We have considered SpOC when OC differentiations were conducted in the absence of osteoclastogenic factor RANKL. In such condition, BCPs derived monocytes showed higher OC differentiation than HVs monocytes (**Figures 2A, B**; **Supplementary Figure 1B**). Parallel differentiations carried out in RANKL presence resulted in higher OC numbers after differentiation of both BCPs and HVs derived monocytes, being significantly only for HVs (**Figure 2B**). RANKL addition promoted higher OC number increase percentage in HVs (268.46%) compared to BCPs (91.96%).

In addition, SpOC cultures from BCPs showed larger OC with more nuclei compared to HVs cultures, both in the presence or absence of RANKL (**Figures 2C, D**). Addition of RANKL only significantly increased number of nuclei per OC in both groups when compared to SpOC condition (**Figure 2D**).

**Figure 3:**
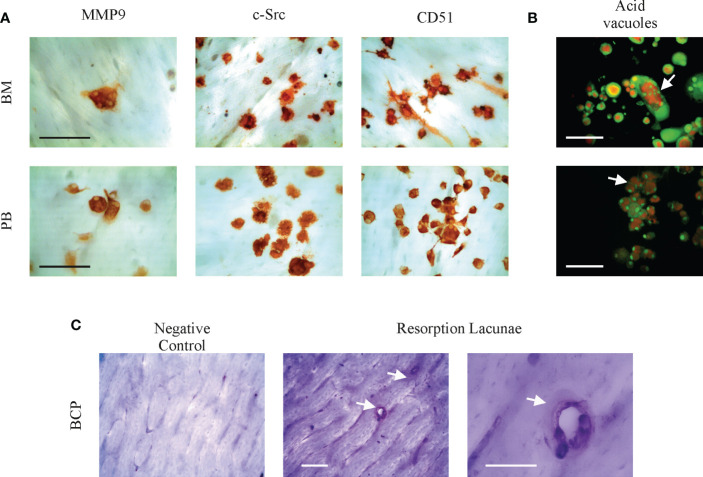
Bone marrow (BM) and peripheral blood (PB) spontaneously derived osteoclasts (OCs) are mature and functional. **(A)** Representative pictures showing cell surface antigens MMP-9, c-Src and αV integrin (CD51) identifying mature OC cultured from a breast cancer patient (BCP) on bovine cortical bone slices by immunocytochemistry. Scale bars: 100 µm. **(B)** Arrows show resorption lacunae acidification (acid vacuoles) on BCP derived OC by Acridine Orange staining. Acridine Orange is green at neutral pH and red at acidic pH. Scale bars: 100 µm. **(C)** Representative pictures showing BCP-BM derived OC resoption lacunae formation on bovine cortical slices. After OC removal, cortical bone slices were stained with Toluidine Blue; arrows indicate resoption lacunae. Scale bars: 100 µm.

SpOC *in vitro* observed in BCPs derived precursors both from BM and PB becomes physiologically relevant if OC maturation and functionality is proven. **Figure 3A** show expression of mature OC markers MMP-9, c-Src and CD51 (αV integrin), in both, BCPs and HVs derived SpOC cultures performed on cortical bone slices. OC functionality was assessed by acidification lagoons visualization using Acridine Orange and resorption lacunae formation on bone slides (**Figures 3B, C**; **Supplementary Figures 2A, B**).

Altogether these results indicate a pre-activated state of BM-MNCs and PB monocytes to derive OCs in BCPs. SpOC could reach a mature phenotype and showed to be functional, suggesting their bone resorption potential *in vivo.*

### 3.2 Characterization of OCPs from BM and PB

**Figure 4:**
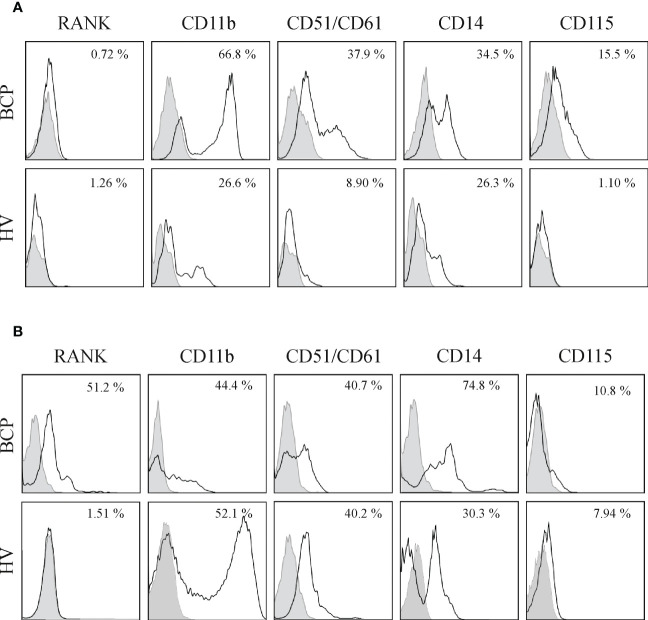
Bone marrow (BM) and peripheral blood (PB) derived mononuclear cells (MNCs) from breast cancer patients (BCPs) express osteoclast precursor’s (OCPs) markers. **(A)** Representative flow cytometry histograms of BM-MNCs surface antigens from a BCP and a healthy volunteer (HV). Percentage represents positive cells (□) with respect to isotype control (▪). **(B)** Representative flow cytometry histograms of PB-MNCs surface antigens from a BCP and a HV. Percentage represents positive cells (□) with respect to the isotype control (▪).

SpOC observed in BCPs cultures may result from isolated MNCs pre-commitment towards OC lineage. Therefore, we performed a phenotypic profiling based on key surface markers involved in the osteoclastogenesis process: RANK, CD51 (alphaV)/61(beta3), CD11b, CD14 and CD115 (24, 27). FACS analysis performed on freshly isolated BM derived MNCs showed significantly higher percentage of cells expressing CD11b and CD14 in BCPs compared to HVs, thus, indicating a greater commitment of hematopoietic progenitors towards monocytic lineage (**Table 1** and **Figure 4A**). Monocyte committed progenitors showed OC bias as indicated by higher yet not significant percentage of cells expressing RANK, CD51/61 and CD115 (**Table 1** and **Figure 4A**). In turn, freshly isolated PB-MNCs showed a significantly higher percentage of cells expressing RANK in BCPs compared to HVs (**Table 1** and **Figure 4B**).

**Table 1:** BM-MNCs and PB-MNCs phenotypic profile.

BM-MNCs				PB-MNCs		
Markers (%)	BCPs (n=4) vs HVs (n=8)		p	BCPs (n=5) vs HVs (n=5)		p
RANK	2.70 ± 0.84	1.14 ± 0.48	NS	24.72 ± 7.27	3.15 ± 0.68	0.0419^c^
CD51/61	22.07 ± 7.63	10.27 ± 4.23	NS	53.22 ± 14.04	56.63 ± 9.78	NS
CD11b	45.30 ± 6.54	24.51 ± 5.00	0.0485^a^	66.20 ± 6.83	84.79 ± 1.32	NS
CD14	21.83 ± 4.82	7.93 ± 2.37	0.0485^b^	67.47 ± 6.83	66.76 ± 0.87	NS
CD115	4.42 ± 2.78	0.95 ± 0.41	NS	10.66 ± 4.26	11.74 ± 4.18	NS

Data is shown as Mean ± SEM. Statistical analysis. (a, b) non-parametric, Mann Whitney test and (c) Unpaired t-test with Welch’s correction. MNCs, mononuclear cells; BCPs, breast cancer patients and HVs, healthy volunteers; BM, bone marrow and PB, peripheral blood; NS, no statistically significant difference.

Direct analysis of BM and PB-MNCs suggested an *in vivo* pre-commitment towards OC lineage in BCPs.

### 3.3 Key factors regulate SpOC in BCPs

**Figure 5:**
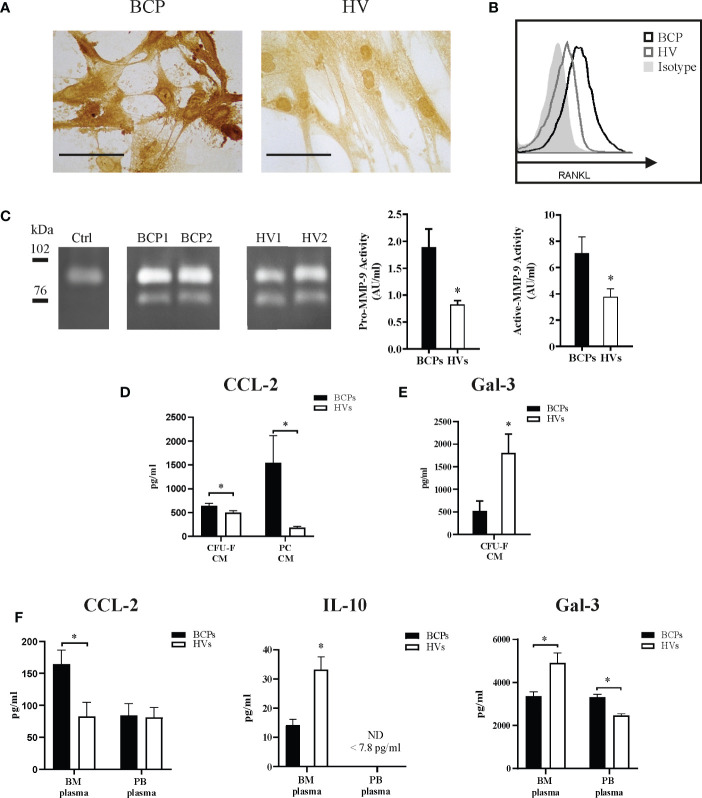
Key factors regulate spontaneous osteoclastogenesis in breast cancer patients (BCPs). **(A)** Representative immunocytochemistry showing membrane RANKL (mRANKL) expression in mesenchymal stem/stromal cells (MSCs) from a BCP and a healthy volunteer (HV). Scale bars: 100 µm. **(B)** Example of flow cytometric analysis showing mRANKL expression in MSCs from a BCP, HV and isotype control. **(C)** Example of Zymography gel showing Pro-MMP-9 and active-MMP-9 enzymatic activity in conditioned media (CM) collected from colony forming units-fibroblastic (CFU-F) assay. Control (Ctrl)= supplemented α medium containing 20% FBS and incubated equally as CM of BCPs and HVs. Bands upper than 90 kDa correspond to pro-MMP-9 and bands under 78 kDa corresponds to active-MMP-9. Right panel shows semi-quantitative densitometric analysis of pro-MMP-9 (92 kDa) and active-MMP-9 (76 kDa). Pro-MMP-9 enzymatic activity (BCPs, n=7 and HVs, n=6); Statistical analysis: parametric, Student’s t-test with Welch correction; p< 0.022(*) and active-MMP-9 enzymatic activity (BCPs, n=7 and HVs, n=7); Statistical analysis: parametric, Student’s t-test; p< 0.035(*). Activity expressed as arbitrary units/ml (AU/ml). **(D)** CCL-2 levels in CFU-F-CM and MSC primary cultures CM detected by ELISA. Data are shown as mean ± SEM. BCPs, n=10 and HVs, n=14. Statistical analysis: parametric, Student’s t-test with Welch correction; p < 0.050(*). **(E)** Gal-3 levels in CFU-F-CM detected by ELISA. Data are shown as mean ± SEM. BCPs, n=8 and HVs, n=8. Statistical analysis: parametric, Student’s t-test with Welch correction; p < 0.050(*). **(F)** CCL-2 levels (BCPs, n=6 and HVs, n=10), IL-10 levels (BCPs, n=6 and HVs, n=6) and Gal-3 (BCPs, n=5 and HVs, n=7) in BM and PB-plasma detected by ELISA. Data are shown as mean ± SEM. Statistical analysis: parametric, Student’s t-test with Welch correction; p < 0.050(*).

Imbalanced patterns in key factors known to regulate OCs differentiation and maturation may explain SpOC observed in BCPs. Accordingly, we investigated RANKL expression and secretion in BM derived MSCs. Membrane bound RANKL (mRANKL) was widely expressed in MSCs from both BCPs and HVs, showing differential expression level per cell when BCPs (n=5, ++++) and HVs (n=5, +++) were compared (**Figure 5A**). We confirmed higher mRANKL expression levels in BCPs MSCs compared to HVs by FACS as exemplified in **Figure 5B**. Additionally, RANKL expression was higher in total MSCs protein lysates from BCPs compared to HVs when evaluated by western blot (**Supplementary Figure 3**). Secreted RANKL levels measured in CM collected from primary sub-confluent MSCs´ cultures by ELISA were under the assay detection limit (≤ 31.25 pg/ml) for both study groups. Together, these results may suggest mRANKL form as one potential regulator of OC differentiation in BM.

We additionally quantified pro-MMP-9 and active MMP-9 levels in CM collected from BCPs and HVs CFU-F assays as indirect osteoclastogenic factor *via* proteolytic degradation of the extracellular matrix components which are important OCP chemo-attractants and differentiation factors. No differences were observed between groups (**Table 2**).

**Table 2:** Pro-MMP-9 and active MMP-9 levels in CM of CFU-F.

Levels (ng/ml)	BCPs (n=10) vs HVs (n=14)		p
Pro-MMP-9	10.49 ± 2.65	9.02 ± 2.33	NS
Active-MMP-9	7.30 ± 2.38	9.03 ± 2.34	NS

Data is shown as Mean ± SEM. Statistical analysis: nonparametric, Mann Whitney test. BCPs: breast cancer patients and HVs: healthy volunteers. CM, Conditioned media; CFU-F, fibroblast colony-forming unit; NS, not significative.

Although MMP-9 levels were similar for both groups, the activity could be different. Therefore, we semi-quantified MMP-9 gelatinolytic activity as shown in **Figure 5C**. Gelatinolytic activity was specific and calcium dependent, no activity was seen in control media and activity was blocked when calcium was chelated by EDTA. Both pro- and active MMP-9 activity were higher in CM derived from BCPs cultures, suggesting BM-MSCs from BCPs may be more prone to extracellular matrix proteolytic degradation and factors release.

Pro-osteoclastogenic chemokine CCL-2 levels resulted higher in CM collected from CFU-F assays and MSCs´ primary cultures from BCPs compared to HVs (**Figure 5D**). Here, we also report higher CCL-2 levels in BM plasma from BCPs compared to HVs as a direct correlation with SpOC observed in BM-MNCs from BCPs (**Figure 5F**).

Additionally, we measured the osteoclastogenesis suppressors Gal-3 and IL10 in BM plasma. As expected, BM plasma levels of IL-10 and Gal-3 were lower in BCPs compared to HVs (**Figure 5F**). Interestingly, a significant decrease of Gal-3 levels in CM collected from CFU-F assays from BCPs was observed when compared to HVs (**Figure 5E**).

PB-SpOC observed in BCPs raised the question whether circulating cell profiling and PB plasma can be used as a surrogate of BM niche to address the initiation of bone metabolism imbalance processes *in vivo*. As shown in **Figure 5F**, key factors suggested to be indicators of SpOC in BM such as CCL-2 or IL-10 are not relevant or not yet changed in PB. Of note, depending on the *in vivo* compartment studied, same factor may indicate a different process as evident with Gal-3 (**Figure 5F**).

Finally, other pro- (IL-1β, IL-7, IL-17, TNF-α and M-CSF) and anti-osteoclastogenic factors (CCL-5, GM-CSF, IL-4, and INF-γ) (28–34) levels were quantified in BM and PB plasma for both BCPs and HVs samples. No remarkable differences were observed (data not shown). In addition, no significant differences of IL-7, M-CSF, CCL-5, IL-6, IL-8 and IL-11 levels were found in the CM of CFU-F assays (14 days) from BCPs compared to HVs (data not shown).

Altogether these results depict a complex regulation network of the SpOC process observed in BCPs. Some key factors at BM level are promising candidates to evaluate bone metabolism imbalance at early stages, before bone resorption markers increase systemically.

### 3.4 Mature miRs and SpOC in BCPs, alternative predictive markers

**Figure 6:**
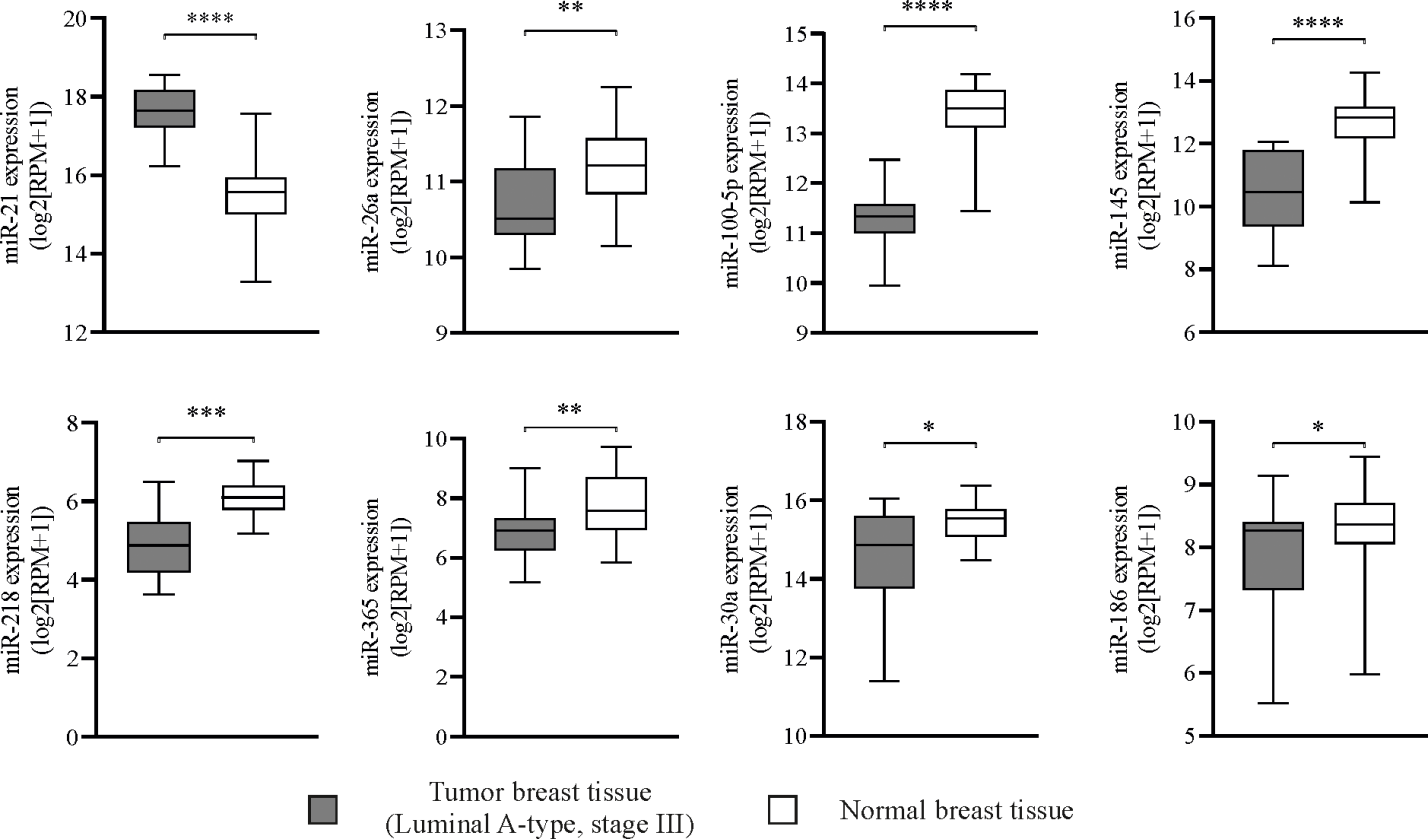
*Mature miRNAs regulate spontaneous osteoclastogenesis in breast cancer patients (BCPs), alternative predictive markers.* Box plots showing miRNA (miR) expression levels in primary tumor samples from patients with luminal A-type invasive ductal breast carcinoma, stage III (n=16) and normal breast tissues (n=56) from TCGA datasets. Statistical analysis: parametric, Student’s t-test with Welch correction; p<0.05 (*), p<0.005 (**), p<0.001 (***) and p<0.0001 (****). Tumor breast tissue (▪) and normal breast tissue (□).

miRs expression in primary tumor tissue from BCPs was compared to normal breast tissue. Only miR-21 expression, a positive regulator of OC differentiation, was increased in tumor tissue compared to normal breast tissue (**Figure 6**). Negative regulators of osteoclastogenesis such as miR-26a, miR-100-5p, miR-145, miR-218, miR-365, miR-30a and miR-186 showed significantly decreased expression in tumor tissue compared to normal breast tissue (**Figure 6**). We evaluated expression levels of key factors CCL-2, RANKL and MMP-9 and found higher expression of RANKL and MMP-9 in tumor tissue compared to normal breast tissue (**Supplementary Figure 4A**). Interestingly, CCL-2 expression appeared decreased in tumor tissue compared to normal breast tissue (**Supplementary Figure 4A**). No statistic correlation was observed between breast tumor tissue miR-21 expression levels and those of CCL-2, RANKL and MMP-9, probably due to the low number of samples (**Supplementary Figure 4B**). Normal breast tissue with a higher “n” robustly showed no correlation between miR-21 and CCL-2 or RANKL expression levels, however, MMP-9 expression showed a positive correlation with that of miR-21 (**Supplementary Figure 4B**).

## 4 Discussion

Target organs for metastasis are not random, cancer cell organotropism relies on molecular mechanisms which are still not fully understood (13). As we commented before citing Stephen Paget hypothesis, breast cancer cell (“seed”) colonization and growth in BM (“soil”) depends on the local microenvironment (35, 36). We have previously described that BM microenvironment in advanced BCPs prior to bone metastasis show specific aspects which are favorable for cancer cell extravasation and colonization (37). We have previously shown that BM of BCPs displays lower MSCs self-renewal together with a deficient osteoblast differentiation, unable to support proper bone formation and suggesting an imbalance in bone metabolism which may favor cancer cells establishment and growth (14, 15). Although BM metastasis is considered a subtype of bone metastasis, evidence suggests that the BM involvement in metastatic spread may represent a pre-condition for bone metastasis in which BM components “prepare/develop” the PMN in the bone (38).

In the present work, we show that advance luminal A- type BCPs without BM/bone metastasis present SpOC in BM and PB, complementing the picture of bone metabolism imbalance in the BM-PMN of these patients. Mature OC hallmarks include fusion processes visualized by multinucleation, which has a direct relationship with resorptive activity, positive TRAP staining and the presence of proteases, such as cathepsin K and MMP-9, and expression of c-Src which mediate attachment to bone matrix, OC migration over bone surface and sealing zone formation that delineates the bone resorbing compartment (24, 39). BM-MNCs of BCPs, including myeloid progenitors and monocytes, differentiated *in vitro* directly into OCs showing most of the maturation hallmarks above mentioned.

VitD3 is a known osteoclastogenic factor which induces RANKL production in VitD3 receptor expressing cells such as MSCs, osteoblasts and osteocytes (40–42). Even though, VitD3 receptor has been reported to be expressed in PB derived monocytes, it is mostly used *in vitro* to stimulate BM-MNCs osteoclastic differentiation by directly acting on MSCs and osteoprogenitors (43–49). Surprisingly, culture supplementation with VitD3, only slightly increased OC differentiation in HVs cultures and affected even reversely BCPs SpOC. This observation may imply a saturation effect, probably a maximum *in vitro* differentiation capacity was reached by BCP samples. Saturation could be explained by full *in vivo* pre-activation of OCPs and/or by the fact that BCP samples contained lower MSCs/osteoblasts numbers, as we have previously shown, and being those the main responders to VitD3 signal for RANKL production, VitD3 effect is hampered (15).

Interestingly and in line with several works showing OC differentiation priming outside BM during inflammatory processes linked to diseases like rheumatoid arthritis, osteoporosis or periodontitis, PB derived monocytes from BCPs showed higher SpOC differentiation efficiency (10, 21). SpOC correlation observed between BM and PB uncovers a promising non-invasive surrogate system to evaluate on going bone resorption processes in BCPs.

An impressive characteristic observed mostly in BM *in vitro* SpOC was the faster pace in which OCPs fused and showed maturation properties in BCPs compared to HVs. Such observation prompts us to study the pre-activated state of OCPs in BCPs. Before seeding BM isolated MNCs or PB derived monocytes for *in vitro* SpOC, part of the cells were interrogated by FACS for a panel of surface markers: RANK, CD51/61, CD11b, CD14 and CD115 (c-fms). Studies on murine models have classified as early OCs whose expressing c-fms+/RANK- which turn into late OCP *via* M-CSF stimulation and subsequently differentiate in OC in the additional presence of RANKL (50). In BCPs both markers tend to be higher expressed in BM, being RANK significantly higher in monocytes from PB. We have previously shown higher levels of RANKL in PB plasma from untreated advanced BCPs (stage III); thus PB-OCPs (RANK+) could be responsible in part for the observed peripheral SpOC (37). In BM derived cells from BCPs, CD14 and CD11b markers are higher expressed than in HVs evidencing not only an activation of OCPs but also suggesting the putative identification of myeloid derived suppressor cells (6, 51). Further evidence in this direction has to be collected, however, the possibility of promoting a hospitable bone microenvironment for tumor cells both by shutting anti-tumor immune response and releasing growth factors *via* bone resorption would denote a key finding in the maintenance of the “vicious cycle”. The OC marker vitronectin receptor αvβ3 appears in late OCPs mediating OC adhesion to the bone matrix (52). We show that in BM from a reduce number of BCPs, CD51/61 is higher expressed than in HVs meanwhile in PB, values from both groups are comparable. This result is consistent with the faster *in vitro* maturation of SpOC derived from BM. The small panel marker we showed here denotes a proof-of-principle for the utility of such an approach to indicate risk of future bone metastasis. As an example, integrin αVβ3 the cognate receptor of vascular cell adhesion molecule 1 (VCAM-1) and OCP marker could be a good complement for the BM marker panel, since we have previously shown significant higher levels of VCAM-1 in BM plasma from untreated advanced BCPs compared to HVs (37, 53).

OCPs activation and subsequent SpOC observed in BCPs may be promoted by osteoclastogenic factors produced at BM or primary tumor sites. Thus, we have studied key factors which may be involved in the process. RANK/RANKL interaction is the definitive trigger for OC differentiation (54, 55). RANKL protein is expressed as a membrane-bound and as secreted protein, being the later a result of either proteolytic cleavage or alternative splicing of the membrane form (56). *In vitro* works suggested mRANKL to be more potent than the soluble form in activating OCs and most recently Honma et al. revealed transmembrane form of RANKL as major contributor to the induction of mature OC under physiological conditions in an *in vivo* murine model (57–59). We have previously shown that BCPs and HVs have similar levels of soluble RANKL in BM plasma (37). Here we showed that BCPs derived MSCs, which are in close contact with BM-MNCs, like hematopoietic progenitors, present higher mRANKL expression than MSCs from HVs. Together, these results indicate that OC differentiation and maturation rely on cell-to-cell interactions in BCPs BM niche. Interestingly, higher RANKL expression could be associated with previous studies from our group, where we found significantly lower levels of the anti-osteoclastogenic factor OPG in CM from BM derived CFU-F cultures of untreated advanced BCPs when compared to HVs (37, 60).

Recently, it has been shown that Dickkopf-Related Protein 1 (Dkk-1) secreted by the breast cancer cells circulates to BM/bones where it attracts OCPs to form a PMN by suppressing the LRP5–ß-catenin–Rnasek axis (61). Our group has previously reported higher levels of Dkk-1 in PB plasma from untreated advanced BCPs compared to HVs even before bone metastasis was observed, which could partially explain the increase number of OCPs in BM of BCPs (15).

Following up the importance of MSCs, as key component of the BM environment and regulators of bone formation/resorption processes, we report increased MMP-9 activity in supernatants from BCPs derived CFU-F cultures when compared to HVs. MMP-9 are proteolytic enzymes which degrade extracellular matrix components and are widely expressed by MSCs and OCs in the BM (62, 63). OC chemo-attractants, like vascular endothelial growth factor (VEGF) and TGFß, are released from the non-mineralized bone matrix by MMP-9 (64–67). In addition, TGFß acts directly on hematopoietic cells to enhance the OC formation (68). MMP-9 has also been shown to degrade preform IL-1β into a soluble active form which mediates OC differentiation and potentiated OC maturation and survival (69–71). Therefore, MMP-9 activity in BM microenvironment may complement key events that enhance OC processes in BCPs.

Additionally to MMP-9, we have measured CCL-2 levels in BM plasma and CFU-F and MSCs primary culture supernatants as indicator of monocyte and OCPs chemotaxis by CCR2 (receptor of CCL-2) binding, and OC pro-survival and pro-differentiation factor (72–75). In BCPs BM compartment, CCL-2 levels were higher than in HVs. However, in this study we have not registered higher CCL-2 levels in PB plasma from BCPs, contrary to observations made by Lubowicka et al. which related CCL-2 to breast cancer progression (76).

Therefore, we suggest our observations in BM at this stage of the disease depict a local scenario prompt to mediate myeloid cells recruitment, OCP activation and differentiation and perhaps tumor cells chemotaxis (13, 77).

Gal-3 a member of the lectin family, was recently added to the bone metabolism equation. It was found to be expressed both in osteoblasts and OCs, and shown to promote osteoblast differentiation by positive regulation of the transcription factor RUNX2 and stabilization of ß-Catenin levels (78–80). Simon et al. proposed Gal-3 as a novel mechanism for osteoblasts to control OC differentiation and to maintain trabecular bone homeostasis independently of the RANKL/OPG-axis (80). Gal-3, which can be modified by MMP-9 activity, was lower in BM plasma and CM of CFU-F assay from BCPs (81). This result indicates a lack of suppressive effect or control on OC differentiation in BM from BCPs. Here once more, results obtained in BM compartment should be considered and interpreted separately from observations in PB. Higher levels of Gal-3 were registered in PB plasma from BCPs, which were in line with observations from Topcu et al. (82). This last group found that serum Gal−3 levels were significantly higher in BCPs and did not significantly differ according to clinical and tumor characteristics or metastatic disease in the patients (82). The importance and exact role of circulating Gal-3 in BCPs is still to be determined.

Besides canonical OC differentiation stimulation based on RANKL and M-CSF signaling, alternative pathways linked to inflammatory cytokines have been described (8, 9, 83, 84). Combination of bone metabolism regulation cytokines produced by osteoblast and osteocytes together with evidence of RANKL production by T-cells defined the new term `osteoimmunology´ to describe the crosstalk between the immune system and osteoclastogenesis (10, 75–77). In this regard, we have explored several cytokines as potential SpOC markers in BM and PB plasma from BCPs. Here we report decreased levels of anti-inflammatory cytokine IL-10 in BM plasma from BCPs. IL-10 is considered an anti-osteoclastogenic cytokine, it downregulates cytokines like TNF-α, IL-1, and IL-6 which support bone resorption, it inhibits NFATc1 expression and its nuclear translocation and promotes OPG expression (28, 85–88).

Finally, we evidenced the potential influence of distant primary tumor on bone biology aspects by investigating miRs expression levels in tumor tissue from BCPs. Recent studies which focused on controlling excessive bone resorption with novel approaches have identified several miRs involved in OCs development and activity (7, 16–18). Tumor derived miRs could act on nearby cells (autocrine or paracrine) or distant cells (endocrine) through traversing the circulation *via* exosomes transfer, making possible a distant regulation of the primary tumor on the PMN (89, 90). Based on TCGA datasets, we described here up regulation of miR-21 in tumor tissue from luminal A-type advanced BCPs. miR-21 is considered an onco-miR since it is expressed in serum of many cancer patients and it has been correlated with cancer progression and OC differentiation by e.g. NFATc1 promotion amongst other mechanisms (18, 91). Accordingly, Asgari et al, reported that serum exosomal miR-21 levels in breast cancer luminal B-type patients considerably increased compared to healthy individuals, highlighting the potential interest of studying miR-21 specifically in breast cancer (92). In parallel, we report downregulation of miR-26a, miR-100-5p, miR-145, miR-218, miR-365, miR-30a and miR-186 in tumor tissue from luminal A-type advanced BCPs, all anti-osteoclastogenic miRs as it is well summarized in Weivoda et al. (18). These authors described that miR-218 negatively modulates OC differentiation by several mechanisms, meanwhile miR-365 inhibits MMP-9 and Cathepsin K modulating bone resorption and miR-26a inhibits DCSTAMP protein expression, critical for OC fusion (18). In addition, gain of function studies with miR-100–5p suppressed *in vivo* bone resorption by targeting FGF21 and miR-145 “agomir” inhibits OC activity in mice by lowering Smad3 expression (93). Finally, miR-30a attenuates osteoclastogenesis *via* inhibition of DCSTAMP/FOS/NFATc1 signaling pathways and miR-186 negatively regulates Cathepsin K to modulate osteoclastic bone resorption (94, 95).

## 5 Conclusions

In the present work we show for the first time SpOC occurring in BM and reflected systemically in untreated advanced luminal A-type BCPs prior to bone (micro)-metastasis appearance. This is a unique set up to determine early bone metabolism imbalances and key factors affecting it before abnormalities in classical bone turn over markers (ALP, P1NP, CTX-1, NTX-1, etc.) are seen. To date, there is no way to alert BCPs´ risk to develop BM/bone metastasis and take action by strict follow up and preventive treatment. Our work shows such an alert can be detected and sets the bases for further and deeper studies to define robust prognosis biomarkers.

## Data availability statement

The original contributions presented in the study are included in the article/**Supplementary Material**, further inquiries can be directed to the corresponding author.

## Ethics statement

The studies involving human participants were reviewed and approved by Comité de Ética “Dr. Enrique T. Segura”. Instituto de Biología y Medicina Experimental (IBYME). Buenos Aires, Argentina. The patients/participants provided their written informed consent to participate in this study.

## Author contributions

VFV and FRB carried out the experiments and analyzed data, conceptualized and wrote the manuscript, reviewed and edited the manuscript during the whole revision process. LMM performed part of the experiments and analyzed the data. MBG, bioinformatic data extraction and analysis, reviewed and edited the manuscript during the whole revision process. HC contributed reagents/materials and reviewed the manuscript. FD, LF, RHB, and EB collected the samples and confirmed the diagnosis, revised and approved the final manuscript. AMC-T contributed reagents and analyzed the data of pro and active MMP-9 ELISA, reviewed the final manuscript. NAC, conceptualization, writing - review and editing, supervision, and funding acquisition. All authors contributed to the article and approved the submitted version.

## Supplementary material

Supplementary Figure 1 Bone marrow (BM) mononuclear cells (MNCs) and peripheral blood (PB) CD14+ monocytes spontaneous and induced differentiation into osteoclasts (OCs). **(A)** Representative pictures showing TRAP staining performed on spontaneously (Sp) and induced (10^-8^ M VitD3) differentiated OC derived from BM of a breast cancer patient (BCP) or a healthy volunteer (HV) at day 18 of culture. TRAP + multinucleated OC (arrows) are shown. Scale bars: 100 µm. **(B)** Representative pictures showing TRAP staining performed on spontaneous and induced (25ng/ml M-CSF and 25ng/ml RANKL) differentiated OC derived from CD14+ monocytes of a BCP or a HV PB-CD14+ monocyte cultures, at day 18. Arrows show TRAP+ multinucleated OC. Scale bars: 100 µm.

Supplementary Figure 2 Bone marrow (BM) and peripheral blood (PB) derived osteoclasts (OCs) from healthy volunteers (HVs). **(A)** Arrows show resorption lacunae acidification (acid vacuoles) on a HV derived OC by Acridine Orange staining. Acridine Orange is green at neutral pH and red at acidic pH. Scale bars: 100 µm. **(B)** Representative pictures showing HV-BM-MNCs derived OC resoption lacunae formation on bovine cortical slices. After OC removal, cortical bone slices were stained with Toluidine Blue; arrows indicate resoption lacunae. Scale bars: 100 µm.

Supplementary Figure 3 Study of RANKL expression in mesenchymal stem/stromal cells (MSCs) from primary cultures by Western Blot. **(A)** RANKL expression in total MSCs protein lysate from breast cancer patients (BCPs) and healthy volunteers (HVs) by Western Blot. **(B)** Raw data showing absolute optical density (O.D) values of bands depicted in panel A. **(C)** Semi-quantification of RANKL in BCPs and HVs. Each sample was normalized to respective ß-Actin control. Statistical analysis: parametric, Student’s t-test; p< 0.020 (*).

Supplementary Figure 4 Study of microRNA (miR)-21, CCL-2, RANKL and MMP-9 in primary tumor samples from breast cancer patients (BCPs) and normal breast tissue. **(A)** miR-21 expression levels and gene expression levels of CCL-2, RANKL, MMP-9 in breast tumor samples from patients with luminal A-type invasive ductal breast carcinoma, stage III (n=16) and normal breast tissues (n=56) from TCGA datasets. Statistical analysis: parametric, by Student’s t-test with Welch correction, p=0.0339 (*) and p<0.0001 (****). **(B)** Correlation of miR-21 expression vs CCL-2, RANKL and MMP-9 expression in breast tumor samples from patients with luminal A-type invasive ductal breast carcinoma, stage III (n=16) (Spearman K=0.1265, p=0.6405; Spearman K=-0.4353, p=0.0937; Spearman K=0.4824, p=0.0606; respectively) and normal breast tissue (n=56) (Spearman K=0.1974, p=0.1447; Spearman K=0.09331, p=0.4940; Spearman K= 0.5405, p<0.0001****; respectively) from TCGA datasets. Statistical analysis: Spearman’s rank correlation coefficient. Tumor breast tissue (●) and normal breast tissue (□).
