# Supplementary figures and images for: Spontaneous Osteoclastogenesis, a risk factor for bone metastasis in advanced luminal A-type breast cancer patients
Source: Front Oncol. 2023 Feb 20;13:1073793. doi: 10.3389/fonc.2023.1073793 (PMC9986318; doi:10.3389/fonc.2023.1073793)

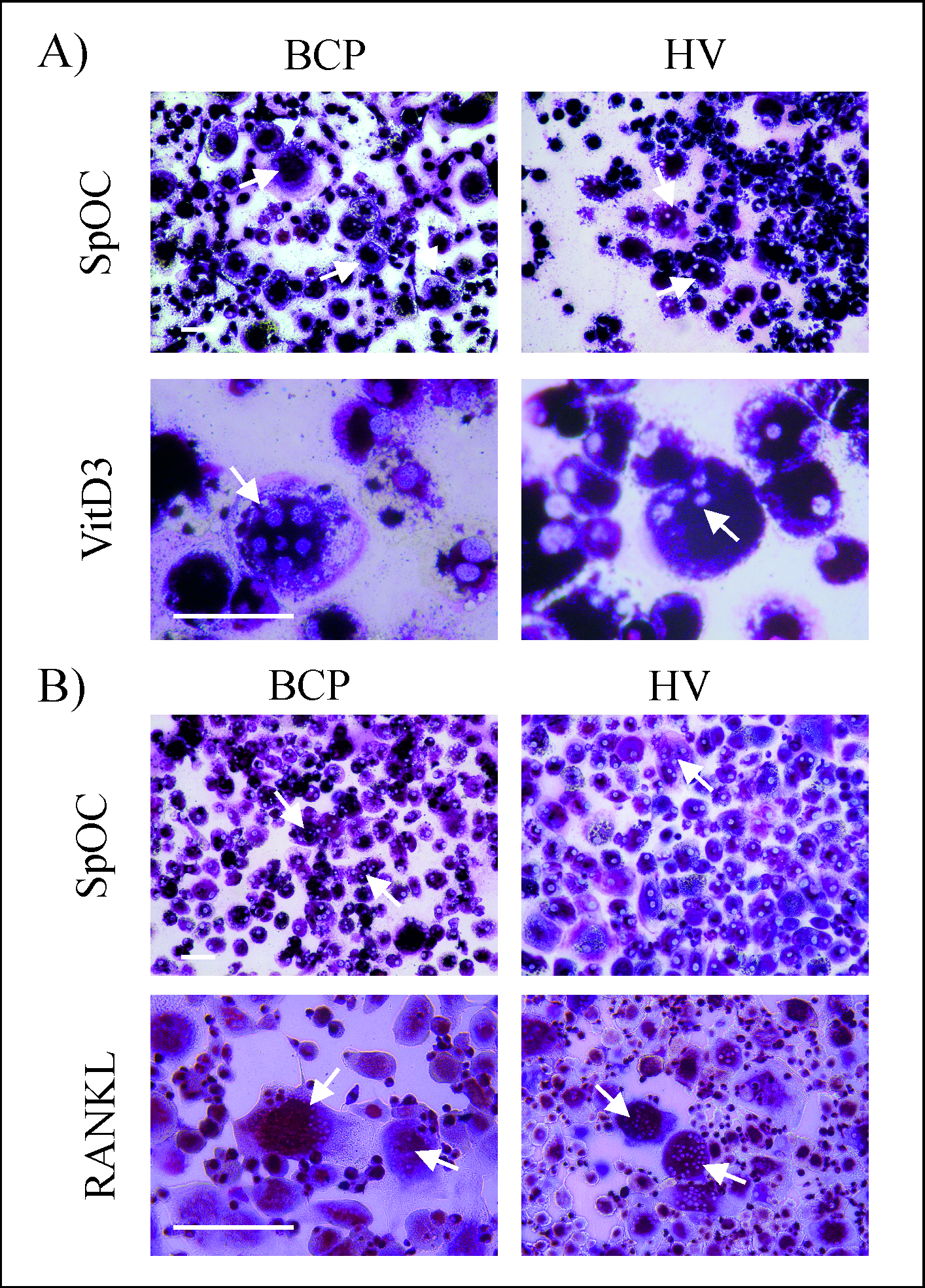

Supplement: Supplementary Figure 1 — Bone marrow (BM) mononuclear cells (MNCs) and peripheral blood (PB) CD14+ monocytes spontaneous and induced differentiation into osteoclasts (OCs). (A) Representative pictures showing TRAP staining performed on spontaneously (Sp) and induced (10-8 M VitD3) differentiated OC derived from BM of a breast cancer patient (BCP) or a healthy volunteer (HV) at day 18 of culture. TRAP + multinucleated OC (arrows) are shown. Scale bars: 100 µm. (B) Representative pictures showing TRAP staining performed on spontaneous and induced (25ng/ml M-CSF and 25ng/ml RANKL) differentiated OC derived from CD14+ monocytes of a BCP or a HV PB-CD14+ monocyte cultures, at day 18. Arrows show TRAP+ multinucleated OC. Scale bars: 100 µm. [file Image_1.tif]

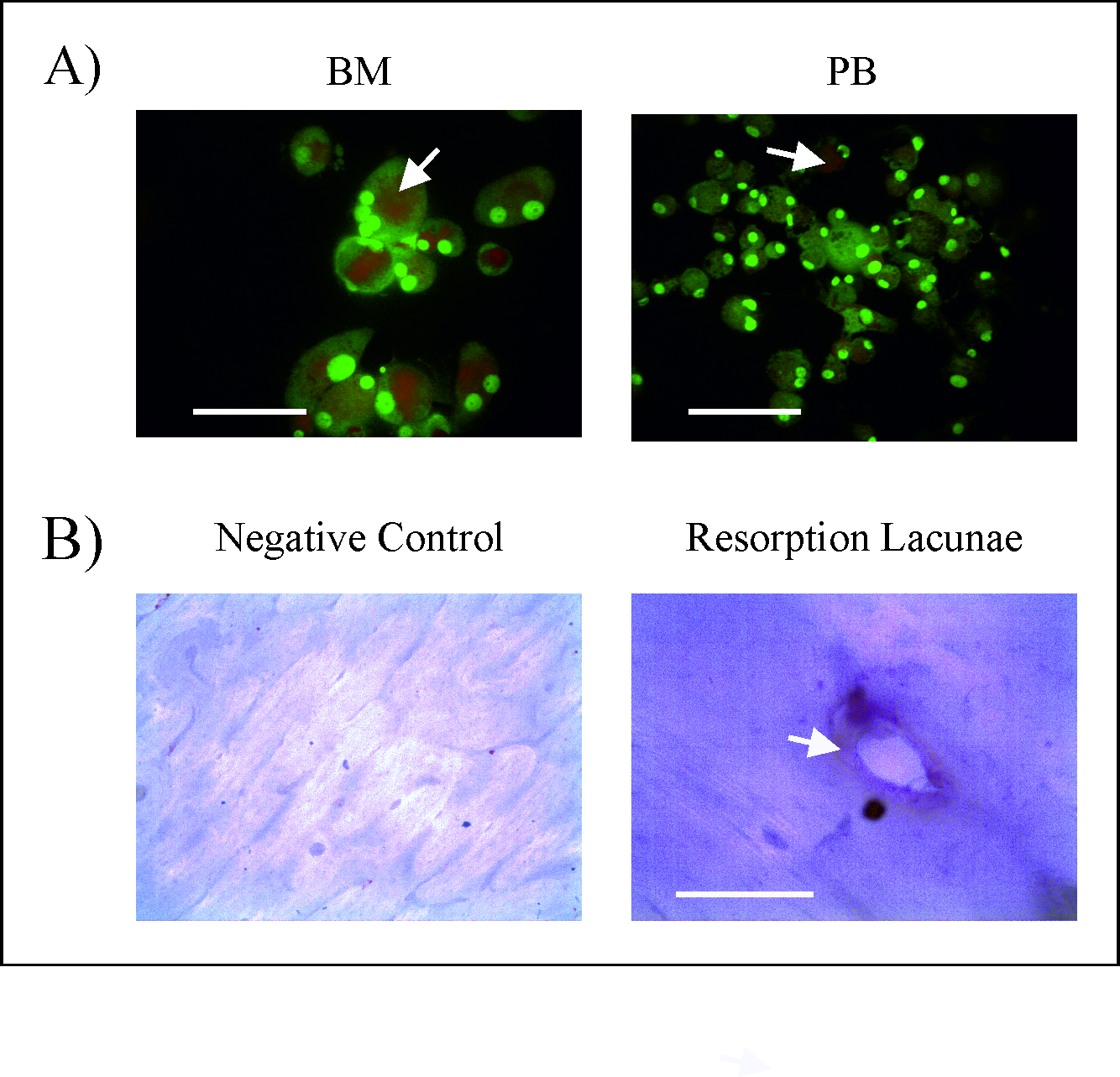

Supplement: Supplementary Figure 2 — Bone marrow (BM) and peripheral blood (PB) derived osteoclasts (OCs) from healthy volunteers (HVs). (A) Arrows show resorption lacunae acidification (acid vacuoles) on a HV derived OC by Acridine Orange staining. Acridine Orange is green at neutral pH and red at acidic pH. Scale bars: 100 µm. (B) Representative pictures showing HV-BM-MNCs derived OC resoption lacunae formation on bovine cortical slices. After OC removal, cortical bone slices were stained with Toluidine Blue; arrows indicate resoption lacunae. Scale bars: 100 µm. [file Image_2.tif]

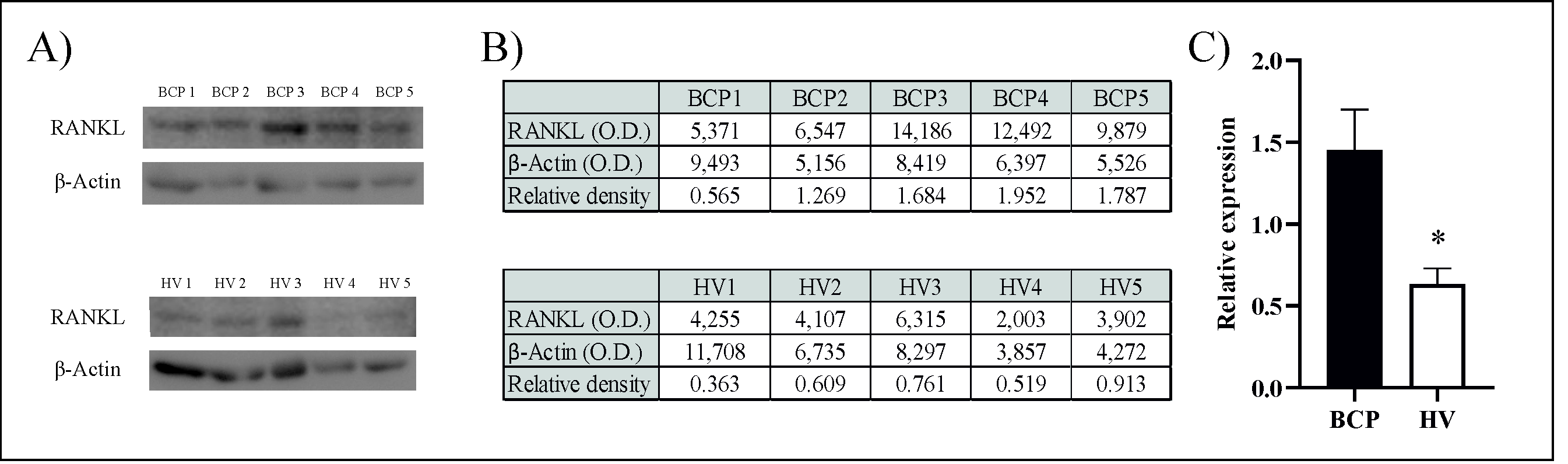

Supplement: Supplementary Figure 3 — Study of RANKL expression in mesenchymal stem/stromal cells (MSCs) from primary cultures by Western Blot. (A) RANKL expression in total MSCs protein lysate from breast cancer patients (BCPs) and healthy volunteers (HVs) by Western Blot. (B) Raw data showing absolute optical density (O.D) values of bands depicted in panel A. (C) Semi-quantification of RANKL in BCPs and HVs. Each sample was normalized to respective ß-Actin control. Statistical analysis: parametric, Student’s t-test; p< 0.020 (*). [file Image_3.tif]

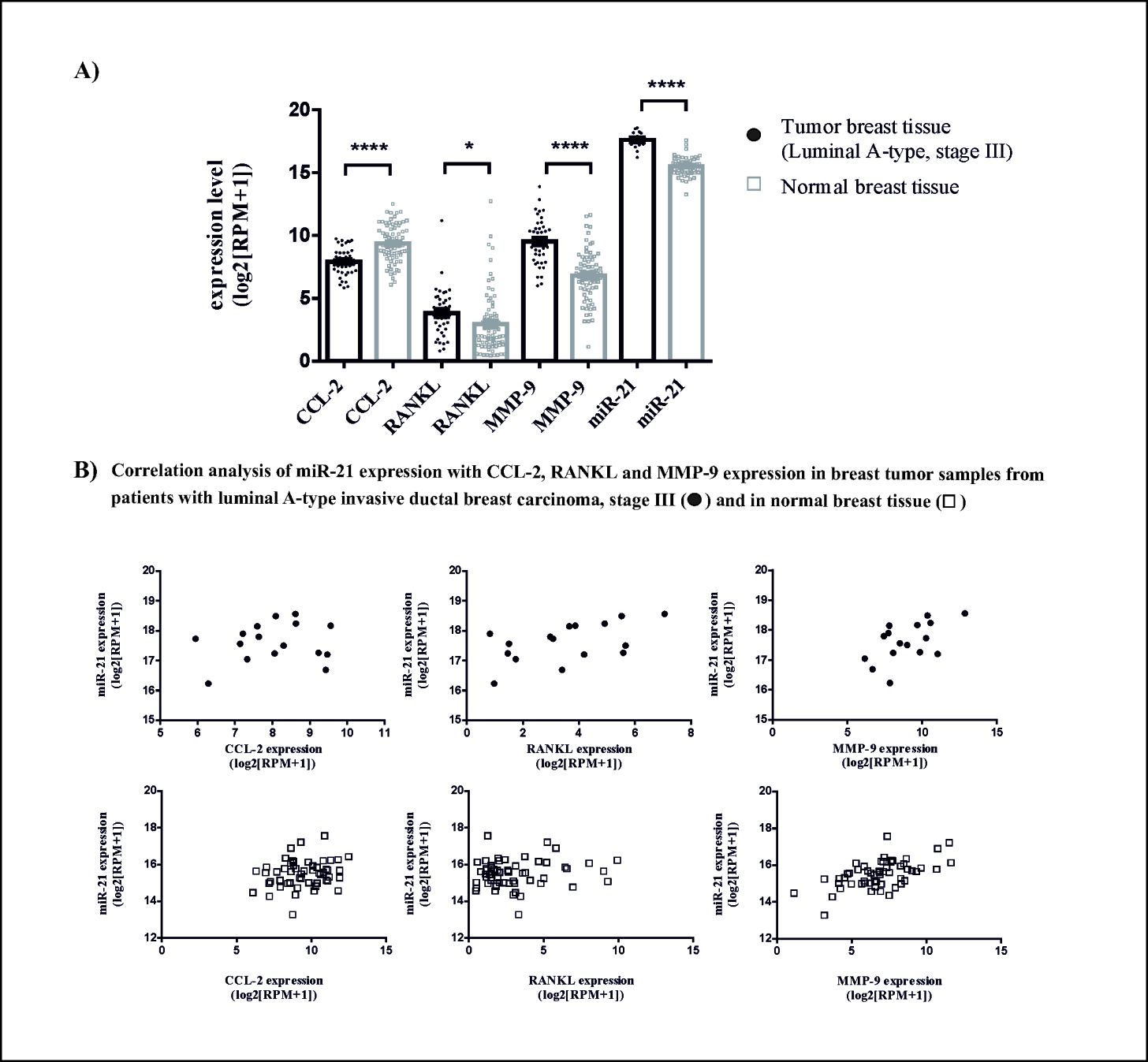

Supplement: Supplementary Figure 4 — Study of microRNA (miR)-21, CCL-2, RANKL and MMP-9 in primary tumor samples from breast cancer patients (BCPs) and normal breast tissue. (A) miR-21 expression levels and gene expression levels of CCL-2, RANKL, MMP-9 in breast tumor samples from patients with luminal A-type invasive ductal breast carcinoma, stage III (n=16) and normal breast tissues (n=56) from TCGA datasets. Statistical analysis: parametric, by Student’s t-test with Welch correction, p=0.0339 (*) and p<0.0001 (****). (B) Correlation of miR-21 expression vs CCL-2, RANKL and MMP-9 expression in breast tumor samples from patients with luminal A-type invasive ductal breast carcinoma, stage III (n=16) (Spearman K=0.1265, p=0.6405; Spearman K=-0.4353, p=0.0937; Spearman K=0.4824, p=0.0606; respectively) and normal breast tissue (n=56) (Spearman K=0.1974, p=0.1447; Spearman K=0.09331, p=0.4940; Spearman K= 0.5405, p<0.0001****; respectively) from TCGA datasets. Statistical analysis: Spearman’s rank correlation coefficient. Tumor breast tissue (●) and normal breast tissue (□). [file Image_4.tif]
